# Predicting IQ change from brain structure: A cross-validation study

**DOI:** 10.1016/j.dcn.2013.03.001

**Published:** 2013-03-15

**Authors:** C.J. Price, S. Ramsden, T.M.H. Hope, K.J. Friston, M.L. Seghier

**Affiliations:** Wellcome Trust Centre for Neuroimaging, UCL, London, UK

**Keywords:** IQ change, Neuroimaging, Circular inference, Biased sampling, Non-independent errors, Cross-validation

## Abstract

Procedures that can predict cognitive abilities from brain imaging data are potentially relevant to educational assessments and studies of functional anatomy in the developing brain. Our aim in this work was to quantify the degree to which IQ change in the teenage years could be predicted from structural brain changes. Two well-known *k*-fold cross-validation analyses were applied to data acquired from 33 healthy teenagers – each tested at Time 1 and Time 2 with a 3.5 year interval. One approach, a Leave-One-Out procedure, predicted IQ change for each subject on the basis of structural change in a brain region that was identified from all other subjects (i.e., independent data). This approach predicted 53% of verbal IQ change and 14% of performance IQ change. The other approach used half the sample, to identify regions for predicting IQ change in the other half (i.e., a Split half approach); however – unlike the Leave-One-Out procedure – regions identified using half the sample were not significant. We discuss how these out-of-sample estimates compare to in-sample estimates; and draw some recommendations for *k*-fold cross-validation procedures when dealing with small datasets that are typical in the neuroimaging literature.

## Highlights

• We quantify how well IQ changes in teenagers can be predicted from brain scans.
• We compare different ways to cross-validate predictions from neuroimaging.
• We demonstrate the advantage of using Leave-One-Out cross-validation.
• We illustrate the limitations of using IQ as a measure of cognitive potential.

## 1 Introduction

Neuroimaging data are most commonly used to find brain areas where the functional response, or structural measurement, can be predicted by experimental, behavioural or demographic variables. In this case, the mapping of interest is from behavioural measurements (the independent variable) to brain measurements (the dependent variable) and enable one to infer that certain brain areas are associated with the experimental manipulation. These inferences can then be empirically tested with new data. For example, after demonstrating that the right cerebellum was activated during verbal fluency tasks (Petersen et al., 1988, Petersen et al., 1989), the same authors reported that damage to the right cerebellum impaired verbal fluency (Fiez et al., 1992). In other words, a functional imaging study of healthy participants predicted functional specialisation that was confirmed with a neuropsychological (structural imaging) study of patients, which led to a clinically relevant conclusion.

Inferences about cognitive abilities from brain imaging data have also been made in the developmental context. For example, Hoeft et al. (2007) predicted children's reading skills from a combination of behavioural and neuroimaging measures. Cross-validation procedures of the kind reported in Hoeft et al. are essential in this context, because predictions about behaviour will only generalise if they apply to subjects that were not used to select the brain region used to make those predictions. In cross validation procedures, one sample is used to identify brain regions mediating the behavioural phenotype, and another sample is used to predict the behavioural phenotype using those regions. If the same subjects are used in both steps, the predictive validation is circular (non-independent) and there is no replication of the structure-function relationships. This is referred to as “circularity”, “double dipping”, “the non-independence problem” or “biased estimates” (Kriegeskorte et al., 2009, Poldrack and Mumford, 2009, Vul et al., 2009).

In the current study, we illustrate the use of two different cross-validation procedures, with the aim of quantifying how much of the variance in IQ change, measured over the teenage years (Ramsden et al., 2011, Ramsden et al., 2012), can be predicted from structural brain changes – when the predictions for IQ change are made for subjects that did not contribute to the selection of predictive brain areas (i.e., the region selection and prediction process used independent data). The validation approaches we report, “Leave-One-Out” and “Split-half”, are commonly used to test whether the results of a statistical analysis generalise to an independent sample, and they are especially useful when new samples are costly or difficult to collect.

There are many *re-sampling* techniques that we could have adopted in this context, including bootstrap, jack-knife, permutation tests and cross-validation. The “Split half” and “Leave-One-Out” procedures we assess here are both variants of *k*-fold cross validation. They involve partitioning the full set of data into *k* non-overlapping samples or sets: *k* − 1 samples are used as training sets (e.g., to generate a hypothesis/model) and the remaining sample is used as a validation set (e.g., to test a hypothesis/model). In the present context, this translates to *k* − 1 samples being used to identify brain areas associated with a behavioural phenotype (behaviour-to-brain) and the remaining sample being used to predict behaviour from brain measurements (brain-to-behaviour). This procedure is then repeated with different training and validation sets (derived from the same overall sample) – and the results from each iteration/fold are averaged to produce a single estimate. The advantage of repeating the procedure (with *k* folds) is that all observations are used for both training and validation, without replacement. More specifically, *k*-fold cross validation only assumes that the original sample is chosen at random from the population and the samples (partitions or subsets) are in turn chosen at random from that original sample. Although the validation and training sets are drawn from the same population, cross-validation is not considered to produce biased results (Efron and Tibshirani, 1997, Hastie et al., 2009).

Variations in the *k*-fold procedure differ according to how the full sample is partitioned and with the number of iterations used (i.e., the value of *k*); see review in Arlot and Celisse, 2010. The robustness and appropriateness of each *k*-fold procedure can be assessed against several criteria; including bias, variance, sensitivity, completeness and computational cost. Different *k*-fold procedures are expected to perform comparably when the sample is relatively large; however, differences may emerge when the sample size is too small (e.g., in the case of a biased or skewed distribution). Ideally, different procedures can be tested with increasing values of *k* (varying between 2 to the number of subjects); however, the computational cost can become unmanageable; particularly when many iterations must be performed for a given *k* value. In the current paper, we compared *k*-fold cross-validation when the value of *k* was set to either its lower limit (*k* = 2 = the Split-half analysis) or to its upper limit (for our sample size *k* = 33 = the Leave-One-Out analysis). These *k* values reflect the two extremes for the given number of subjects (i.e., half of the subjects for *k* = 2 to all but one subject for *k* = 33). The effectiveness of the two procedures can then be compared on the basis of: (i) type-II errors during region selection, (ii) the proportion of variance in measured IQ change that could be accounted for by structural change, when tested on the remaining (independent) subsets, and (iii) the computational cost of both procedures.

In more detail, to implement the Leave-One-Out approach, all but one of the available observations are used in the training set and the remaining observation (that is left out) is used to validate the results (Hastie et al., 2009). The procedure is then repeated *k* times, with *k* being equal to the number of observations in the full sample, and with each observation occurring once in the test set and *k* − 1 times in the training set. The advantages of this approach are that (i) power in the training set is maximised (by including all but one observation) and (ii) there are a (usually comparatively small) finite number of splits that is equal to the number of observations, see Efron and Tibshirani (1997), Hastie et al. (2009), Strother et al. (2002) for further discussion. The Leave-One-Out procedure should therefore be efficient (statistically speaking) for small sample sizes.

To implement the Split-half analysis, the full sample is split in half by randomly assigning data to two sets (*A* and *B*), so that both sets are of (approximately) equal size. In this 2-fold or Split-half cross-validation, training starts on *Set A*, with testing on *Set B*, followed by training on *Set B* and testing on *Set A*. The main disadvantage of the Split-half approach is that the ‘training sets’ (*Set A* in the first iteration, and *Set B* in the second) are smaller than they could be. Put simply, if the power per sample is low, then small training set sizes could reduce the sensitivity of detecting effects for subsequent validation in the test set. As discussed in Kohavi (1995), when the sample is small – in the context of a small *k* value (here *k* = 2) – there is variance due to the random effects of the training sets themselves (Kohavi, 1995). One solution is to average the results after repeating the procedure with multiple two-way splits. However, if the overall sample size is too small, none of the training analyses will have sufficient power to detect effects of interest (Poldrack and Mumford, 2009). Moreover, for a reasonably sized sample, there will be an almost infinite number of possible partitions of the same data. If only a few random partitions are tested, some observations may never be selected in the validation subsample, whereas others may be selected more than once. These considerations suggest that – for a maximally sensitive analysis – the Leave-One-Out procedures may be preferable over split half procedures. In what follows, we test this conjecture quantitatively, using a reanalysis of previously reported data.

Our data were from a longitudinal study of verbal and performance IQ (henceforth VIQ and PIQ) in teenagers. We have already used these data (Ramsden et al., 2011) to show that the change in VIQ and PIQ over a 3.5 year period significantly predicted changes in local grey matter density over the same time period (henceforth Time 1 to Time 2). We have also reported a brief addendum to this finding (Ramsden et al., 2012) that used one iteration (partition) of a Split-half cross-validation procedure (Hastie et al., 2009) to show the reverse; i.e., that changes in brain structure predicted changes in IQ. The current paper provides a more in depth exploration of two *k*-fold cross-validation procedures that can be used to test the validity of such predictions, given independent data. Previous neuroimaging studies have used *k*-fold cross validation procedures to provide unbiased estimates of generalisation in terms of feature selection, model comparison, or classification accuracy. Here, we used cross validation to estimate the out-of-sample effect size when predicting the behaviour of individual subjects from structural brain changes. Given our relatively small sample size (*n* = 33), it is likely that different *k*-fold cross-validation techniques may show different outcomes (e.g., Braga-Neto and Dougherty, 2004, Martens and Dardenne, 1998).

## 2 Methods

This study was approved by the Joint Ethics Committee of the Institute of Neurology and the National Hospital for Neurology and Neurosurgery, London, UK. The data and pre-processing were the same as those used in Ramsden et al. (2011). Brain imaging and behavioural assessments were collected from 33 neurologically normal teenage subjects at two time points in 2004 (Time 1) and 2008 (Time 2).

### 2.1 Subjects

**Fig. 1:**
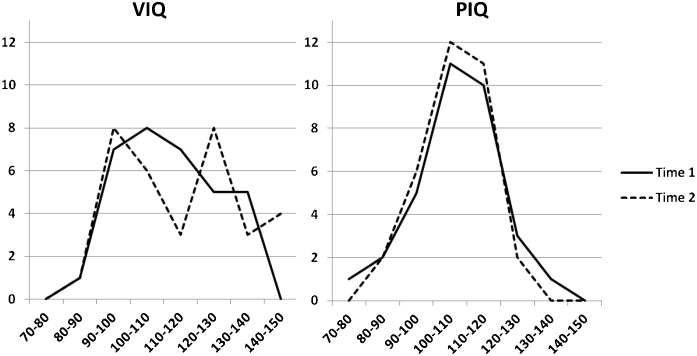
Distribution of VIQ and PIQ scores at Time 1 and Time 2. The plots show the frequency distribution of VIQ and PIQ scores at both test points in ten-point bands (the *y* axis represents the number of subjects in each band). Means (and standard deviations) at Time 1 and Time 2 were: 113 (15.1) and 116 (18.0) for VIQ; and 108 (12.3) and 107 (9.6) for PIQ. The corresponding minimal/maximum were 84–139 and 90–150 for VIQ and 74–137 and 83–124 for PIQ. In all cases, there was no evidence that the distributions differed significantly from a normal distribution using the Shapiro–Wilk statistic (Time 1 VIQ: *p* = 0.470; Time 2 VIQ: *p* = 0.070; Time 1 PIQ: *p* = 0.787; Time 2 PIQ: *p* = 0.355).

The teenagers were selected to provide a range of IQ scores (see Table 1 for details), with a distribution of scores that did not differ significantly from normal (see Fig. 1). The mean age of the subjects was 14.1 years (range = 12–16) at Time 1, and 17.7 (range = 15–20 years) at Time 2. The mean time between Time 1 and Time 2 was 3.5 years, with a minimum of 3.3 years and a maximum of 3.9 years. During the intervening years, there were no testing sessions and subjects (or their carers) were not told that they would be invited back for further testing. On both testing occasions, each subject and their carers gave informed consent. The study was approved by the joint ethics committee of the Institute of Neurology and the National Hospital for Neurology and Neurosurgery, London, UK.

**Table 1:** Behavioural data for each subject.

ID	Time 1		Time 2		Change (Time 2 − Time 1)	
	VIQ	PIQ	VIQ	PIQ	VIQ	PIQ
1	115	110	95	97	−20	−13
2	109	112	95	98	−14	−14
3	136	112	123	114	−13	2
4	115	110	104	111	−11	1
5	127	116	119	98	−8	−18
6	102	94	96	109	−6	15
7	108	109	104	106	−4	−3
8	133	101	130	114	−3	13
9	128	137	125	124	−3	−13
10	98	112	95	102	−3	−10
11	92	96	90	104	−2	8
12	96	116	94	110	−2	−6
13	100	90	100	95	0	5
14	117	125	117	113	0	−12
15	91	97	91	95	0	−2
16	102	119	102	102	0	−17
17	120	97	121	114	1	17
18	127	115	131	111	4	−4
19	137	105	142	107	5	2
20	108	110	113	107	5	−3
21	121	109	128	110	7	1
22	84	74	91	83	7	9
23	98	97	106	100	8	3
24	101	88	110	104	9	16
25	139	115	150	124	11	9
26	131	112	142	114	11	2
27	117	121	128	116	11	−5
28	129	118	144	117	15	−1
29	113	124	130	113	17	−11
30	91	105	108	105	17	0
31	120	103	138	85	18	−18
32	104	101	127	104	23	3
33	110	103	133	117	23	14
Av	112.7	107.7	115.8	106.8	3.1	−0.9
SD	15.1	12.3	18.0	9.6	10.6	10.2

### 2.2 Behavioural testing

IQ was measured using the Wechsler Intelligence Scale for Children (WISC-III) at Time 1 and the Wechsler Adult Intelligence Scale (WAIS-III) at Time 2. It was necessary to use different tests to ensure that the tests were age-appropriate. Raw scores on all tests were converted to age standardised scores (mean 100, standard deviation 15), using procedures in the published statistical manuals for each test – that are based on large samples. Across our sample, there were no significant differences in Time 1 and Time 2 scores for VIQ (113 and 116) and PIQ (108 and 107), see Table 1, and scores at the different time points were highly correlated (VIQ *r* = 0.81, *p* < 0.001; PIQ *r* = 0.59, *p* < 0.001). Nevertheless, within the sample, there was a wide range of score changes between testing points, with some individuals increasing their score and others showing either no change or a fall in score (see Table 1). This within-subject variance ranged from −20 to +23 for VIQ and −18 to +17 for PIQ; with 21% of our sample showing a shift of at least one population standard deviation (15) on the VIQ measure, and 18% on the PIQ measure. It is these changes in IQ that we wanted to predict.

### 2.3 Brain imaging

Scan acquisition used the same equipment and parameters at Time 1 and Time 2: a Siemens 1.5T Sonata MRI scanner (Siemens Medical Systems, Erlangen, Germany) and a T1-weighted Modified Driven Equilibrium Fourier Transform sequence were used to acquire 176 sagittal partitions with an image matrix of 256 × 224, yielding a final resolution of 1 mm^3^ [repetition time/echo time/inversion time = 12.24 ms/3.56 ms/530 ms]. The scan and behavioural tests were carried out on the same day in 56% of cases and within a week of one another in 74% of cases. The maximum interval between testing and scans was 12.9 weeks, with a mean of 1.4 weeks.

### 2.4 Scan processing

Pre-processing of 66 structural images (33 subjects × 2 time points) used SPM8 (http://www.fil.ion.ucl.ac.uk/spm) and the DARTEL toolbox to segment and spatially normalise the brains into a standard template space. Co-ordinates for each voxel were converted to standard MNI space. Normalised grey matter images were generated at 1.5 mm × 1.5 mm × 1.5 mm voxel size and smoothed using an 8 mm isotropic Gaussian kernel at full width half maximum (FWHM). For more details see Ramsden et al. (2011).

### 2.5 Cross-validation methods

Each cross-validation analysis comprised two steps. Step 1 identified regions of interest where grey matter density changed with VIQ or PIQ change (i.e., behaviour to brain); and Step 2 predicted behavioural change in independent subjects – on the basis of grey matter density changes in the regions identified in Step 1 (brain to behaviour). The methodological details of Step 1 were identical for the Split half and Leave-One-Out analysis, the only difference between the analyses was the number of subjects used for each step:

**Step 1:** For all subjects in the region selection stage, the pre-processed images from both time points were entered into a general linear model (ANCOVA), with three covariates that modelled subject specific and time specific effects – to factor out average grey matter density per subject and non-specific (average) changes over subjects with age/time. The first covariate was year of scan, which accounted for any increases or decreases in grey matter density that occur with age. It was entered as minus one for Time 1 and plus one for the Time 2. The second and third covariates were the changes in VIQ and PIQ, respectively: an increase in IQ from Time 1 to Time 2 was encoded as the negative change for Time 1 and the positive change for Time 2. A fall in IQ was entered as the positive change for Time 1 and the negative change for Time 2.

We tested for the effects of VIQ and PIQ change on brain structure using standard procedures in SPM: the effect of VIQ change was identified using contrast weights (0, 1, 0). The effect of PIQ change was identified by the contrast weights (0, 0, 1). In addition, we directly contrasted the VIQ change and PIQ change regressors (0, 1, −1) and (0, −1, 1). Regions of interest for VIQ change and PIQ change were identified using a statistical threshold of *p* < 0.05 corrected for multiple comparisons across the whole brain (in height and/or extent); and where there was also a difference between VIQ change and PIQ change (*p* < 0.01 uncorrected).

**Step 2:** For each subject that was not included in Step 1, grey matter density measurements at Time 1 and Time 2 were extracted from voxels that showed a significant effect of IQ change in Step 1. The difference in grey matter density at Time 1 and Time 2 is referred to as the “*measured grey matter density change*”. These values were then used to predict IQ change. In this way, we were able to calculate the proportion of measured IQ change that could be predicted from brain structure. The following sections explain how the analysis differed for the Leave-One-Out and Split half procedures.

#### 2.5.1 Leave-One-Out analysis

**Fig. 2:**
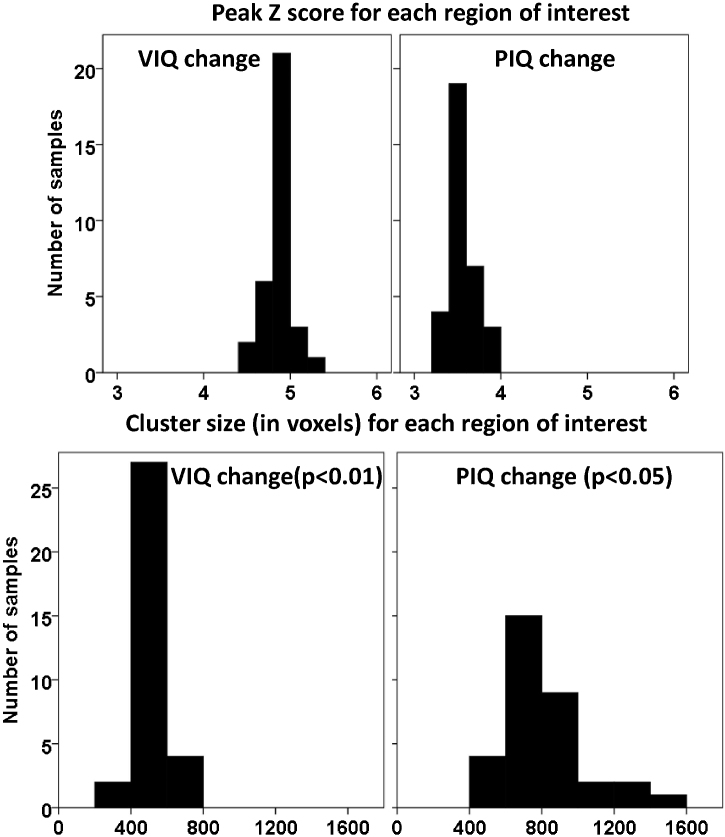
Leave-One-Out procedure – Step 1 results. Upper panel summarises the significance of the effect size at the peak voxels associated with VIQ change and PIQ change. Lower panel summarises the size of the clusters (in voxels) associated with VIQ change and PIQ change. The threshold selected for cluster size is lower for PIQ change (*p* < 0.05 uncorrected) than VIQ change (*p* < 0.01 uncorrected). However, the size of all but two of these clusters reached significance after correction for multiple comparisons across the whole brain (*Z*-score > 4.7), see Table 2 for details.

**Fig. 3:**
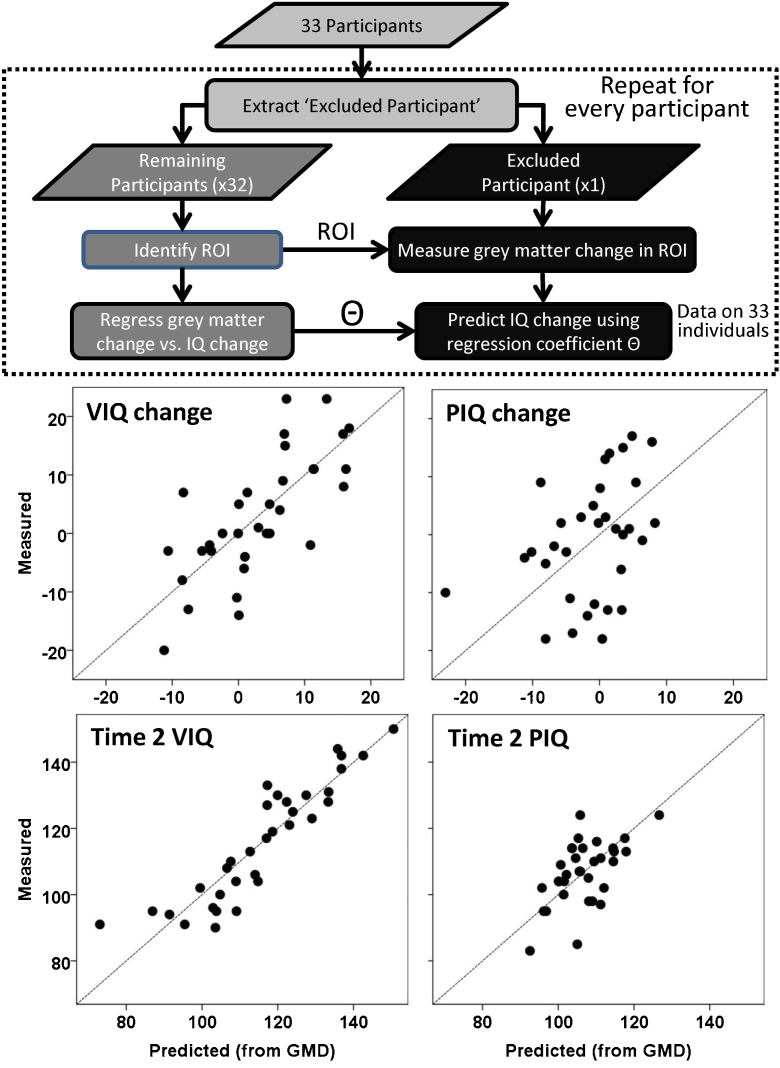
Leave-One-Out procedure and Step 2 results. Upper panel summarises the procedures (see text for details). ROI = region of interest identified in Step 1. Lower panel shows the results of the standard regression analyses (in SPSS) that illustrate the relationship between predicted and measured (i) VIQ change; (ii) PIQ change, (iii) Time 2 VIQ; and Time 2 PIQ. In each case, the predicted values were based on measured grey matter density (GMD) change at the peak voxel identified in Step 1. The Leave-One-Out analysis makes independent predictions for each individual (both the region and the regression parameters are independent of the individual). This contrasts to the Split-half analyses that use independent data to identify the region, but the within sample data to estimate the regression parameters.

Step 1 was conducted 33 times, for each possible combination of 32 subjects. In other words, every subject contributed 32 times to the region selection sample or training set and once to the validation sample or test set, making the cross-validation complete or exhaustive. In total, there were 66 different clusters (VIQ and PIQ for each of the 33 analyses); see Fig. 2. For each Step 1 analysis (with 32 subjects), we calculated the regression slopes that best explained the relationship between measured changes in grey matter density and measured changes in IQ. This regression slope was used to predict IQ change in the excluded subject (Step 2), on the basis of their measured grey matter density change. Predicted IQ change for all 33 subjects was then regressed against their measured IQ change (using standard regression in SPSS) – so that we could calculate the proportion of variance in measured IQ change that could be predicted from brain structure change alone. Likewise, we calculated the proportion of variance in measured Time 2 VIQ/PIQ that could be accounted for when Time 2 IQ was predicted on the basis of measured Time 1 IQ and measured grey matter density change. A summary of the Leave-One-Out procedure is illustrated in Fig. 3.

#### 2.5.2 Split-half analysis

First, we split the subjects in half according to their full scale IQ score. We then randomly assigned half the higher IQ subjects to Group A and the other half to Group B. Likewise; we randomly assigned half the lower IQ subjects to Group A and the other half to Group B. Our rationale here follows the same principle as the *stratified* cross-validation method where subgroups are not random but stratified so that they contain approximately the same proportions of key labels (i.e., high and low IQ) as the original/full set (Delen et al., 2005, Kohavi, 1995). Put another way, cross-validation would only yield meaningful results if the training and validation sets are representative of the full set. In total, there were 16 subjects in Group A and 17 subjects in Group B. This was repeated 25 times, resulting in a total of 50 different subgroups (1A, 1B, 2A, 2B up to 25A and 25B).

**Fig. 4:**
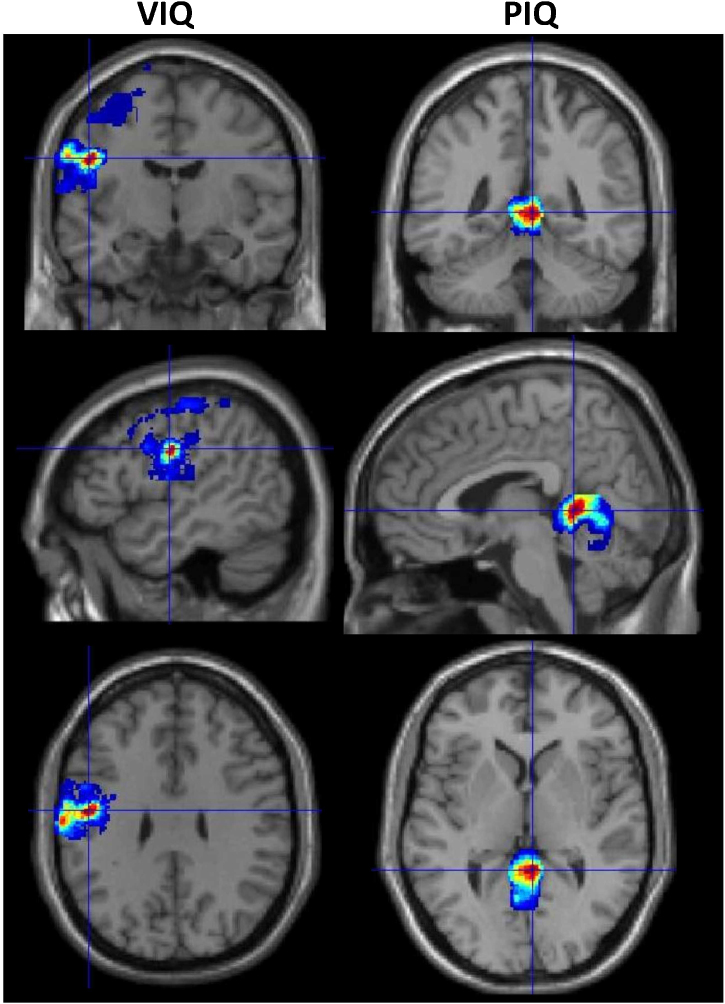
Region Selection – Step 1. The images illustrate the regional clusters selected in Step 1 for the 50 Split half analyses of (A) VIQ change and (B) PIQ change. The colour indicates the number of analyses in which the voxel belonged to a cluster that was selected. The maximum overlap (=45/50 for both VIQ and PIQ) is shown with a blue cross hair positioned at (*x* = −49, *y* = −9, *z* = +30) for VIQ and (*x* = +5, *y* = −45, *z* = +2) for PIQ. Notably, the co-ordinates with the maximum overlap in the Split half analyses corresponded exactly to the co-ordinates of the peak effects (maximum *Z* score) identified in the 33 Leave-One-Out analyses. This demonstrates remarkable consistency in region selection across all the different subsamples.

**Fig. 5:**
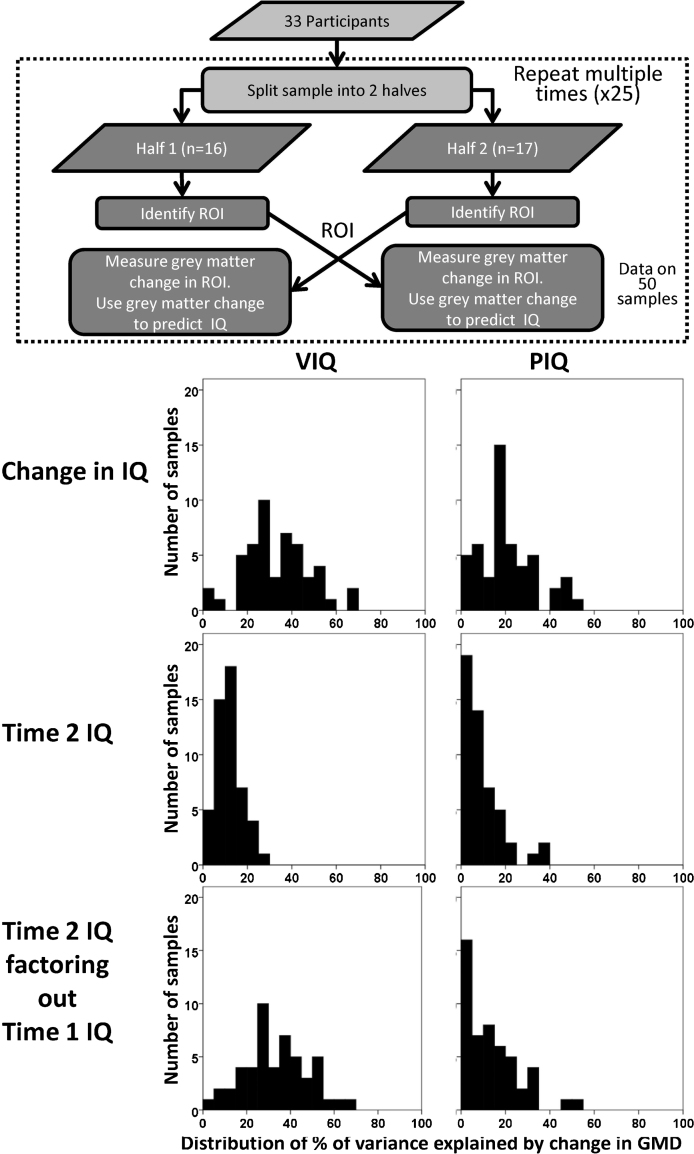
Split-half procedure and Step 2 results. Upper panel summarises the procedures (see text for details). ROI = region of interest identified in Step 1. Lower panel summarises the results with histograms showing the number of analyses (maximum = 50) where *R*^2^ accounted for 0–100% of the variance in (i) Change in IQ, (ii) Time 2 IQ; and (iii) Time 2 IQ after factoring out the influence of Time 1 IQ. The key point to note is that there is substantial variance in the results of the different partitions. This is a consequence of inefficient selection of the ROI when the analysis only includes half the data.

Step 1 was conducted 50 times, once for Group A and once for Group B in each of the 25 splits of the data. The voxels identified for VIQ and PIQ in each analysis were compared by generating an image indexing the frequency with which a voxel entailed a significant effect in each analysis (Fig. 4). In Step 2, the SPSS regression analyses predicted how well the measured grey matter density change (the independent variable) predicted (i) measured IQ change and (ii) measured Time 2 IQ. The regression predicting Time 2 IQ also included Time 1 IQ as an independent variable, and was conducted in a hierarchical fashion, with Time 1 IQ as the first independent variable, and the measured change in grey matter density entered as the second independent variable: this allowed an assessment of the effect of grey matter density changes after removing the effect of Time 1 IQ. An identical procedure was reported in Ramsden et al., 2011, Ramsden et al., 2012 but with different measurements of grey matter density change. However, unlike Ramsden et al. (2011), the grey matter density changes used in the current analyses were extracted from regions that were identified in an independent sample of subjects; and unlike Ramsden et al. (2012), the regressions were repeated 50 times rather than twice. A summary of the Split-half procedure is illustrated in Fig. 5.

## 3 Results

### 3.1 Leave-One-Out analysis

**Step 1:** There was remarkable consistency in the region selection stage of the Leave-One-Out analysis. All 33 analyses found an effect of change in VIQ in the left motor cortex; and an effect of change in PIQ in the anterior cerebellum. Notably, the peak voxel associated with VIQ change in each of the 33 analyses (see Table 2) was identical (*x* = −49, *y* = −9, *z* = +30) to that reported in Ramsden et al. (2011). Moreover, the observed effect sizes were significant (*p* < 0.05 in height) in each of the 33 analyses, after family wise error correction for multiple comparisons across the whole brain. Likewise, the maximum variation in the peak voxel associated with PIQ change (see Table 2) was only 4 mm (*x* = +5 [±2]; *y* = −45 [±2], *z* = +2 [±2]) across all the different analyses. In 31/33 analyses, the effect sizes for PIQ were significant (*p* < 0.05 in extent) after family wise error correction for multiple comparisons across the whole brain. The results of the 33 Step 1 analyses are summarised in Fig. 2. The four histograms show the distribution of (i) *Z* scores for the VIQ peak; *Z* scores for the PIQ peak; cluster size in voxels for the VIQ effect; and cluster size in voxels for the PIQ effect.

**Table 2:** Results of each of the 33 Leave-One-Out analyses.

Subject	VIQ					*V*–*P*	PIQ					*P*–*V*
	Coordinates			*Z* score	#Voxels (*p* < 0.001)	*Z*	Coordinates			*Z*	#Voxels (*p* < 0.01)	*Z* score
	*x*	*y*	*z*				*x*	*y*	*z*			
1	−47	−9	+30	4.9	564	3.4	+4	−45	+1	3.5	762	3.5
	−60	−15	+33	4.4			+6	−42	−6	3.0		
2	−47	−9	+30	4.7	458	3.2	+6	−43	0	3.7	858	3.3
	−60	−15	+33	4.5			−3	−45	+1	3.2		
3	−47	−9	+30	4.8	671	3.4	+6	−43	0	3.7	899	3.1
	−60	−15	+33	4.6			+7	−48	+7	3.2		
4	−47	−9	+30	4.9	504	3.4	+4	−45	+3	4.0	1429	3.1
	−62	−16	+36	4.3			+6	−42	−6	3.6		
5	−47	−9	+30	4.9	507	3.6	+6	−46	+3	3.4	426	2.7
	−60	−15	+33	4.2			+6	−42	−6	2.8		
6	−47	−9	+30	4.7	446	3.3	+6	−46	+3	3.6	819	3.1
	−60	−15	+33	4.3			−3	−45	+1	3.1		
7	−47	−9	+30	4.8	471	3.5	+6	−43	0	3.4	431	2.7
	−60	−15	+33	4.2			+7	−48	+7	3.0		
8	−47	−9	+30	4.9	526	3.4	+6	−46	+3	3.6	980	2.7
	−60	−15	+33	4.3			−3	−45	+1	3.1		
9	−47	−9	+30	4.9	150	3.6	+6	−43	0	3.5	1334	2.6
	−62	−16	+36	4.0	173		+4	−49	+7	3.5		
10	−47	−9	+30	4.7	119	3.2	+6	−43	0	3.6	771	3.0
	−63	−15	+30	4.2	308		−2	−48	+4	3.2		
11	−47	−9	+30	4.9	602	3.5	+6	−43	0	3.6	767	2.7
	−60	−15	+33	4.5			+7	−48	+7	3.2		
12	−47	−9	+30	4.9	526	3.5	+6	−43	0	3.6	945	2.7
	−62	−16	+36	4.4			−3	−45	+1	3.1		
13	−47	−9	+30	4.8	465	3.2	+7	−43	+1	3.5	568	2.6
	−60	−15	+33	4.2			+7	−46	+10	3.0		
14	−47	−9	+30	4.9	515	3.3	+6	−46	+3	3.6	890	2.9
	−60	−15	+33	4.3			+6	−42	−6	3.3		
15	−47	−9	+30	4.9	463	3.3	+6	−46	+3	3.5	654	2.6
	−65	−16	+30	4.2			−3	−45	+1	3.1		
16	−47	−9	+30	4.9	523	3.5	+6	−46	+3	3.7	818	2.8
	−60	−15	+33	4.2			−3	−45	+1	3.1		
17	−47	−9	+30	5.2	573	3.8	+6	−43	0	3.6	635	2.7
	−60	−15	+33	4.2			−3	−45	+1	3.1		
18	−47	−9	+30	4.9	481	3.4	+6	−43	0	3.7	860	2.8
	−60	−15	+33	4.2			−3	−45	+1	3.2		
19	−47	−9	+30	5.1	600	3.5	+4	−45	+1	3.6	721	2.7
	−62	−13	+31	4.4			−6	−49	+6	3.0		
20	−47	−9	+30	4.9	499	3.4	+4	−45	+1	3.7	825	2.7
	−60	−15	+33	4.2			+6	−42	−6	3.1		
21	−47	−9	+30	4.6	361	3.5	+3	−45	+3	3.9	1287	2.5
	−63	−15	+30	3.6			+6	−42	−6	3.2		
22	−47	−9	+30	5.3	540	3.4	+6	−43	0	3.5	693	2.7
	−60	−15	+33	4.2			−3	−45	+1	3.1		
23	−47	−9	+30	4.9	533	3.4	+3	−45	+3	3.6	780	2.7
	−60	−15	+33	4.3			+6	−42	−6	3.1		
24	−47	−9	+30	4.9	531	3.4	+4	−45	+1	3.5	654	2.7
	−63	−15	+30	4.2			+6	−42	−6	3.1		
25	−47	−9	+30	5.0	517	3.5	+6	−46	+3	3.5	748	2.7
	−60	−15	+33	4.3			−3	−45	+1	3.1		
26	−47	−9	+30	4.9	501	3.4	+6	−43	0	3.6	736	2.7
	−60	−15	+33	4.2			+7	−48	+7	3.2		
27	−47	−9	+30	4.9	529	3.4	+4	−46	+3	3.7	1054	2.8
	−62	−16	+36	4.3			+6	−42	−6	3.0		
28	−47	−9	+30	5.1	640	3.7	+3	−45	+3	3.6	775	2.7
	−63	−16	+30	4.7			−6	−49	+6	3.0		
29	−47	−9	+30	4.9	417	3.6	+6	−43	0	3.5	675	2.7
	−60	−15	+33	4.1			+7	−48	+7	3.2		
30	−47	−9	+30	4.9	528	3.2	+3	−46	+1	3.3	596	2.4
	−63	−15	+30	4.2			+6	−42	−6	2.7		
31	−47	−9	+30	4.5	408	3.1	+6	−43	0	3.9	1076	3.1
	−60	−15	+33	4.0			−3	−45	+1	3.2		
32	−47	−9	+30	4.9	518	3.7	+6	−46	+3	3.3	647	2.6
	−60	−15	+33	4.3			−6	−49	+6	2.9		
33	−47	−9	+30	5.0	498	3.6	+4	−45	+3	3.7	601	2.8
	−60	−15	+33	4.2			+6	−42	−6	3.0		

*V*–*P* = peak *Z* score for the direct contrast of VIQ and PIQ; *P*–*V* = the reverse.

**Step 2:** Across all 33 subjects, 53% of the variance in measured VIQ change was accounted for by the VIQ change predicted from grey matter change; and 83% of the variance in measured Time 2 VIQ was accounted for by the Time 2 VIQ predicted from grey matter change (see Fig. 3). The corresponding effects for PIQ were: 14% of the variance in measured PIQ change was accounted for PIQ change predicted from grey matter change; and 33% of the variance in measured Time 2 PIQ was accounted for Time 2 PIQ predicted from grey matter change (see Fig. 3).

### 3.2 Split-half analyses

In Step 1, the Split half analysis was unable to identify brain regions that showed a significant effect of VIQ change or PIQ change, after correction for multiple comparisons across the whole brain. This is a consequence of the increased risk of Type II errors (false negatives), when the sample size is reduced (in this case the sample size was nearly half that used for Step 1 in the Leave-One-Out analysis). To illustrate the results of Step 2, we biased the procedures in Step 1 by selecting the most significant contiguous voxels within a large – anatomically defined – search space (of 42,000 voxels). This space included the left motor cortex and anterior cerebellum, as defined by the Automated Anatomical Labelling (AAL) atlas (Tzourio-Mazoyer et al., 2002). All right hemisphere voxels and left hemisphere occipital, temporal and parietal areas were excluded. This constrained the analyses to regions in the vicinity of those reported in Ramsden et al. (2011). With the selection of 2 clusters within this search space (one for VIQ and one for PIQ) for each of the 50 subgroups, there were a total of 100 different clusters. Fig. 4 provides an illustration of the variance/consistency of the voxels selected for VIQ and PIQ across the 50 different subgroups. Although there is variance in the extent of the voxels selected (as illustrated in the colour coding), the maximum overlap across the 50 different analyses for VIQ and PIQ corresponded to the co-ordinates of the peak activation in the full sample reported in Ramsden et al. (2011) and Step 1 in the Leave-One-Out-analysis.

In step 2, across all 50 groups, measured grey matter change in the VIQ area accounted on average for (i) 33% of the variance in VIQ change and (ii) 34% of the variance in Time 2 VIQ (after factoring out Time 1 VIQ). For PIQ, the measured grey matter change accounted for (i) 20% of the variance in PIQ change and (ii) 13% of the variance in Time 2 PIQ (after factoring out Time 1 PIQ). As shown in Table 3, these values, averaged over 50 samples, are lower than those reported in our two previous reports (Ramsden et al., 2011, Ramsden et al., 2012). This is because the voxel selection in Step 1 is relatively inefficient when based on 16 or 17 subjects (as opposed to 33 subjects). Consequently, there was substantial variance in the results of the 50 individual Split-half analyses. For example, R^2^ varied from 0 to 67% for VIQ at Time 2; and 0 to 51% for PIQ at Time 2, see Fig. 5. The point we want to make here is that these estimates are inconsistent across the 50 different analyses. This reflects an inefficient region selection (rather than an absence of an effect), when only half the sample is used.

**Table 3:** Predicting IQ from brain structure.

	Percentage of IQ variance associated with change in GMD					
	VIQ			PIQ		
	Change in VIQ	Time 2 VIQ		Change in PIQ	Time 2 PIQ	
		Overall	After removing Time 1 VIQ^a^		Overall	After removing Time 1 PIQ^a^
In sample (Ramsden et al., 2011)	58%	*20%*	58%	38%	*13%*	20%
Single Split-half (Ramsden et al., 2012)	52%	*16%*	53%	45%	*15%*	29%
25 Split-half analyses	33%	12%	34%	20%	9%	13%

	Percentage of measured IQ associated with predicted IQ			
	VIQ		PIQ	
	Change in VIQ	Time 2 VIQ	Change in PIQ	Time 2 PIQ
Leave-One-Out (33 individuals)	53%	83%	14%	33%

Figures in italics have been previously published.

a Figures after removing Time 1 IQ are derived from hierarchical regressions – see Section 2 for details.

### 3.3 Comparing the results of the Split-half and Leave-One-Out cross-validation analyses

The results for the various analyses summarised in Table 3 are directly comparable for the analyses predicting the change in IQ but not for the analyses predicting Time 2 IQ. For the latter, the Leave-One-Out analysis compares predicted and measured IQ, whereas the in-sample statistics and Split-half analyses report the proportion of variance explained by grey matter variance. Nevertheless, we can compare the analyses directly by considering the total amount of variance in Time 2 IQ that is accounted for by the combination of grey matter density change and Time 1 VIQ. For the in-sample analyses (Ramsden et al., 2011), this is 86% for VIQ and 48% for PIQ. For the Leave-One-Out analyses, the values are 83% for VIQ and 33% for PIQ. For the Split-half analyses, the average value is 77% for VIQ (with a range of 65–90%) and 43% for PIQ (with a range of 11–69%). The Leave-One-Out is computationally more efficient than the Split-half because it allowed all possible (and finite) partitions to be explicitly tested.

## 4 Discussion

This paper addresses the important issue of out-of-sample estimation of effect sizes and generalisation when assessing correlated changes in structure and function in longitudinal studies. Specifically, the aim was to apply cross-validation procedures to quantify how well structural brain changes predict IQ changes in the teenage years. Two well-known cross-validation procedures were used to provide such out-of-sample estimates. As expected, we found that Leave-One-Out cross-validation provided a more accurate and robust characterisation of our data. Although Split-half cross-validation has been recommended for dealing with the problem of circular inference in neuroimaging (e.g., Kriegeskorte et al., 2009, Poldrack and Mumford, 2009, Vul et al., 2009), it did not perform as consistently or efficiently as the Leave-One-Out procedure in this application.

Our results also show that the relationship between structural brain changes and IQ changes is particularly strong for VIQ. For example, the Leave-One-Out analysis predicted 53% of the measured variance in VIQ changes on the basis of grey matter change alone; and 83% of Time 2 VIQ when both grey matter change and Time 1 VIQ were both accounted for. The Split-half analysis also predicted 77% of the variance in Time 2 VIQ. However, this was only after we had restricted the region selection stage to the most significant voxels – within a large anatomically defined search volume that included the left motor cortex and anterior cerebellum – where effects were reported in our previous report (i.e., we biased the region selection to areas identified from the same subjects). It was not possible to use unbiased region identification in the Split half analysis because, when region selection was based on only 16 or 17 subjects, there was insufficient power to locate effects that were significant after a whole brain correction for multiple comparisons (see discussion in Poldrack and Mumford, 2009). Even within our anatomically restricted search, we still have less confidence in the voxels selected by the Split-half approach. This is reflected in the variance in voxel selection across the 50 different Split-half analyses for each IQ measure (see Fig. 4). The selection of regions with low statistical power also leads to inefficient validation – reflected in the inconsistent estimates of the proportion of variance in Time 2 IQ that was accounted for by grey matter change (65–90% for VIQ).

The predictions for PIQ change were also significant but much less so than those for VIQ. In the Leave-One-Out analysis, grey matter density change only explained 14% of the variance in PIQ; and 33% of the variance in Time 2 PIQ (after Time 1 PIQ had been accounted for). Future studies may be able to improve these predictions by including combinations of regions. For example, in the case of VIQ, we know that vocabulary knowledge (one of the VIQ subtests) predicts grey matter density change in the posterior supramarginal gyri (Grogan et al., 2012, Lee et al., 2007, Richardson et al., 2010). We also know that local white matter changes predict reading (Yeatman et al., 2011) and arithmetic (Tsang et al., 2009) skills. If performance on each IQ subtest is associated with unique brain regions (in addition to the common area in the left motor cortex) then factoring in the contribution of multiple regions to multiple sub-processes is likely to improve the overall predictions.

Our cross-validation procedures produced out-of-sample estimates that were not quantitatively compromised, relative to the in-sample predictions reported in Ramsden et al. (2011), see Table 3. A key aspect of our longitudinal design was that we measured within subject changes with two independent measurements of IQ and brain structure at two different time points. In this way, we could account for between subject variance in brain structure and cognitive ability at a single time point. If future studies could control for the many factors that vary across subjects, they may be able to use the same techniques to predict an individual's cognitive performance from brain structure at a single time point. However, it is likely that such analyses will require very high subject numbers – in the region selection stage – to control for between subject variance (i.e., effects of no interest). We avoided between subject variance of no interest by using a within subjects design. At the same time, we maximised variance in the effects of interest by acquiring data from a sample with a wide range of verbal and performance IQ scores. Subsequent studies are likely to be less robust if there is insufficient variance in the abilities or learning capacities of their subjects.

Our results illustrate three points in favour of the Leave-One-Out procedure, when data are only available from a small number of subjects and the effects are small or noisy. The first is at the level of region selection (Step 1), which is efficient because it is based on the largest possible number of subjects in the training (region selection) group. This was reflected by both the consistency and significance of regional effects in Step 1. The second point in favour of the Leave-One-Out approach is that there is a finite number of possible partitions – that is equal to the number of subjects in the sample. In contrast, the number of partitions that are typically used in Split-half procedures is generally very small compared to the total number possible. Third, the Leave-One-Out approach allows us to compare actual and predicted results in a totally unbiased way at the individual subject level.

Overall, we suggest that the Leave-One-Out analysis is the preferred approach for quantifying out-of-sample estimates of effect size using longitudinal data from a small number of subjects as in our study. However, we are not claiming that the Leave-One-Out approach would necessarily be superior to the Split-half approach in other contexts (Shao, 1993). Indeed, the most convincing outcome would be a full replication of our results using a completely new sample of subjects. This would ensure that there was no inherent bias in our data collection (Stonnington et al., 2010). A full replication would, nevertheless, take several years to conduct given the longitudinal nature of the study. The current results are therefore useful for providing increased confidence that future studies should be able to replicate our findings that changes in cognitive performance can be estimated on the basis of change in grey matter density. Future studies may also be able to use the same techniques to estimate behaviour at a single time point on the basis of regional grey or white matter.

The implication of our results for developmental and educational neuroscience are as follows: In the education context, (i) IQ at a single time point is not a reliable measure of long term potential; (ii) if IQ is changing, then it is not an appropriate baseline for measuring the effects of new teaching methods/interventions, because the effect of teaching a new skill (not in the IQ tests) may interact with late/early development on the ability to perform IQ tests; (iii) the neural correlates of specific cognitive abilities, previously thought to remain constant, can be identified by correlating cognitive change with changes in neuronal infrastructure over time. These longitudinal within-subject studies are more sensitive than cross-sectional studies because they are less prone to error variance from the many sources of inter-subject variability. Finally (iv), inferences about cognitive ability, drawn from neuroimaging data require cross-validation – so that predictions from brain imaging are tested in subjects that did not contribute to region of interest selection. Our results illustrate the procedures and relative merits of using Leave-One-Out cross-validation and the limitations of using Split half analyses, when the sample size is small.

## Conflicts of interest

The author wish to confirm that there are no conflicts of interest.
